# Population Differences in Brain Morphology and Microstructure among Chinese, Malay, and Indian Neonates

**DOI:** 10.1371/journal.pone.0047816

**Published:** 2012-10-24

**Authors:** Jordan Bai, Muhammad Farid Abdul-Rahman, Anne Rifkin-Graboi, Yap-Seng Chong, Kenneth Kwek, Seang-Mei Saw, Keith M. Godfrey, Peter D. Gluckman, Marielle V. Fortier, Michael J. Meaney, Anqi Qiu

**Affiliations:** 1 Department of Bioengineering, National University of Singapore, Singapore, Singapore; 2 Singapore Institute for Clinical Sciences, the Agency for Science, Technology and Research, Singapore, Singapore; 3 Department of Obstetrics & Gynaecology, Yong Loo Lin School of Medicine, National University of Singapore, National University Health System, Singapore, Singapore; 4 Department of Maternal Fetal Medicine, KK Women’s and Children’s Hospital, Singapore, Singapore; 5 Saw Swee Hock School of Public Health, National University of Singapore, Singapore, Singapore; 6 Medical Research Council Lifecourse Epidemiology Unit (University of Southampton) and Southampton NIHR Nutrition Biomedical Research Centre, Southampton, United Kingdom; 7 Liggins Institute, University of Auckland, Auckland, New Zealand; 8 Department of Diagnostic and Interventional Imaging, KK Women’s and Children’s Hospital, Singapore, Singapore; 9 Departments of Psychiatry and Neurology & Neurosurgery, McGill University, Montreal, Canada; 10 Clinical Imaging Research Centre, National University of Singapore, Singapore, Singapore; Institute of Psychology, Chinese Academy of Sciences, China

## Abstract

We studied a sample of 75 Chinese, 73 Malay, and 29 Indian healthy neonates taking part in a cohort study to examine potential differences in neonatal brain morphology and white matter microstructure as a function of ethnicity using both structural T2-weighted magnetic resonance imaging (MRI) and diffusion tensor imaging (DTI). We first examined the differences in global size and morphology of the brain among the three groups. We then constructed the T2-weighted MRI and DTI atlases and employed voxel-based analysis to investigate ethnic differences in morphological shape of the brain from the T2-weighted MRI, and white matter microstructure measured by fractional anisotropy derived from DTI. Compared with Malay neonates, the brains of Indian neonates’ tended to be more elongated in anterior and posterior axis relative to the superior-inferior axis of the brain even though the total brain volume was similar among the three groups. Although most anatomical regions of the brain were similar among Chinese, Malay, and Indian neonates, there were anatomical variations in the spinal-cerebellar and cortical-striatal-thalamic neural circuits among the three populations. The population-related brain regions highlighted in our study are key anatomical substrates associated with sensorimotor functions.

## Introduction

Research into early development has yet to fully examine the impact of population and population-related influences on brain morphology and microstructure, despite studies demonstrating population differences in the brains of adults. Establishing norms that are not limited to one population group is ultimately essential in the unbiased study of both normal and abnormal brain development. Having population-specific baselines from which to investigate deviations is especially important in neuropsychology, as the prevalence, severity and rate of diagnosis of neurodevelopmental disorders vary by ethnicity [1]. A more complete multi-population understanding of early brain development is essential in the detection of early vulnerability and the delivery of appropriate intervention and prevention programs.

There is a paucity of studies examining brain morphological differences among healthy populations of different origins in early life. However, substantial variations in brain anatomy have been reported across adults from different populations, including differences in both brain global size and structural volumes assessed using structural magnetic resonance imaging (MRI). The average brain of Korean or Chinese adults is generally shorter in anterior-posterior length and also rounder in shape than the Montreal Neurological Institute 305 (MNI305) [2] and the International Consortium for Brain Mapping 152 (ICBM152) [3] atlases created based on Caucasian adults’ brain. Moreover, volumetric MRI analysis also shows region-specific volume differences in the cerebellum, amygdala, and orbital frontal cortex between the African-Americans and Caucasians [4], and in the widespread frontal and temporal cortical regions, basal ganglia, and midbrain between Chinese and Caucasians [5].

This study presents a large-scale structural MRI and DTI study of the neonatal brain. We aimed to investigate brain morphology and microstructure differences in the Asian community with three historically distinct populations of Chinese, Malay, and Indian neonates born within the same Singaporean hospital. This study provides the first evidence of differences in brain morphology and microstucture among different Asian populations in early life. Moreover, our study also constitutes a normative reference for healthy brain development among Asian neonates.

## Methods

### Subjects

Subjects were drawn from a cohort study of pregnant Asian women aged 18 years and above attending the first trimester antenatal ultrasound scan clinic at the National University Hospital (NUH) and KK Women’s and Children’s Hospital (KKH) in Singapore. The selection criteria for recruitment to the cohort included a requirement that both parents were of Chinese, Malay or Indian ethnic background. Mothers on chemotherapy, psychotropic drugs, including antidepressant or anxiolytic medications, or with Type I Diabetes Mellitus were excluded. The study design and data collection in this cohort was detailed in [6]. The study was approved by Centralized Institutional Review Boards of the Singapore Health Services and Domain Specific Review Board (DSRB) of National Health Care Group. All subjects gave their written informed consent following a complete description of the study.

One-hundred and eighty nine of the eligible mothers agreed to participate in the imaging study and provided informed consent. Birth outcome measures were obtained from hospital records. All the neonates in this study were born at a gestational age of greater than 34 weeks, at birth weights larger than 2000 g, and with APGAR scores greater than 7 (**Table 1**). The family’s socioeconomic status (monthly household income) and prenatal exposures to alcohol (regular alcohol drinking) and tobacco (regular smoking, daily exposure to smoking at home and job) were ascertained using questionnaires during pregnancy. The family’s social economic status was grouped into 5 categories according to the monthly household income. The majority of mothers were free of illnesses and disability; among those with reported illnesses, 4 reported asthma, 3 previous hyperthyroidism, 1 thalessaemia minor and 1 hypertension.

**Table 1:** Demographic information.

		Chinese (n = 75)	Malay (n = 73)	Indian (n = 29)	Test Statistic	p-value
**Gender, male/female (% male)**		38/37 (53.6)	39/34 (50.0)	17/12 (60.0)	χ^2^_2_ = 0.54	0.765
**Birth Weight (grams), mean (standard deviation)**		3128.69 (405.01)	3106.42 (420.61)	3082.29 (409.57)	F_2,174_ = 0.14	0.867
**Body Weight on the MRI day (grams), mean** **(standard deviation)**		3251.97 (410.37)	3211.02 (393.00)	3159.95 (413.83)	F_2,174_ = 0.57	0.564
**Gestational Age (weeks), mean (standard deviation)**		38.93 (1.08)	38.45 (1.16)	38.69 (1.33)	F_2,174_ = 3.10	**0.048**
**Post-gestational Age on the MRI day (weeks), mean (standard deviation)**		40.36 (1.14)	39.87 (1.21)	39.99 (1.39)	F_2,174_ = 3.04	**0.050**
**Monthly Household Income (S$), %**	**≤999** **1000∼1999 2000∼3999** **4000∼5999 ≥6000** **Unreported**	2.79.329.325.322.710.7	2.719.247.924.74.11.37	6.913.848.36.910.313.8	X^2^_8_ = 20.94	**0.007**
**Prenatal Alcohol Exposure, % yes**		4.0	0	0	X^2^_2_ = 4.11	0.128
**Prenatal Tobacco Exposure, % yes**		44.0	69.9	20.7	X^2^_2_ = 17.95	**<0.001**

### MRI Acquisition

At 5 to 17 days of life, neonates underwent fast spin-echo T2-weighted MRI and single-shot echo-planar DTI scans using a 1.5-Tesla GE scanner at the Department of Diagnostic and Interventional Imaging of the KKH. The scans were acquired when subjects were sleeping in the scanner. No sedation was used and precautions were taken to reduce exposure to the MRI scanner noise. A neonatologist was present during each scan. A pulse oximeter was used to monitor heart rate and oxygen saturation through out the entire scans.

The imaging protocols include i) fast spin-echo T2-weighted MRI (TR = 3500 ms; TE = 110 ms; FOV = 256 mm×256 mm; matrix size = 256×256) and ii) single-shot echo-planar DTI (TR = 7000 ms; TE = 56 ms; flip angle = 90°, FOV = 200 mm×200 mm; matrix size = 64×64). For T2-weighted MRI, 50 axial slices with 2.0 mm thickness were acquired parallel to the anterior-posterior commissure line. Two T2-weighted images were acquired per subject. For DTI, 40 to 50 axial slices with 3.0 mm thickness were acquired parallel to the anterior–posterior commissure line. Nineteen diffusion weighted images (DWIs) with b = 600 sec/mm^2^ and 1 baseline with b = 0 sec/mm^2^ were obtained.

Through visual inspection, 186 out of 189 infants had good T2-weighted MRI scans, while 125 infants had good DTI scans, partially because DTI was last acquired. Data for 12 of the 189 infants were excluded in this study due to motion artefacts (n = 3), low APGAR (n = 2), incomplete maternal data (n = 7).

### Structural T2-weighted MRI Analysis

For individual subjects, the average T2-weighted image was computed by aligning the two T2-weighted MRI acquisitions based on an affine transformation in order to increase signal-to-noise ratio. The average T2-weighted MRI images were then skull-stripped (Smith, 2002) and intensity inhomogeneity corrected (Sled et al., 1998) prior to all subsequent analyses.

#### Global brain size and morphology

The global brain size of neonates, including total brain volume (TBV), three dimensional lengths, and anterior- posterior commissural (AC-PC) length, was quantified on the T2-weighted MR images. The TBV was computed as the multiplication of the image resolution and the number of voxels in subject’s average image after removing the skull. The AC-PC length was defined as the Euclidean distance between the centers of the AC and PC. The length of the brain was calculated as the Euclidean distance between the most anterior and the posterior points of the brain along the AC-PC line. The height of the brain was computed as the Euclidean distance between the most superior and the inferior points of the brain along the line perpendicular to and crossing the midpoint of the AC-PC line on the AC-PC sagittal plane. The width of the brain was calculated as the Euclidean distance between the most left and the right points of the brain along the line perpendicular to and crossing the midpoint of the AC-PC line on the AC-PC axial plane. The ratios of length to width, length to height, and height to width were further used to quantify the global morphological shape of the brain.

#### Brain morphological shape

**Figure 1:**
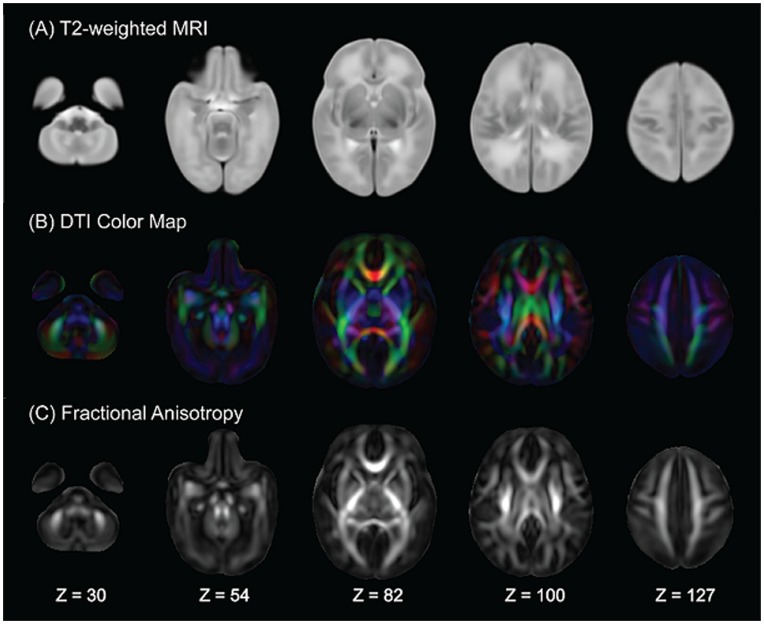
Neonatal structural MRI and DTI atlases. Rows (A-C) respectively show the axial slices of the T2-weighted MRI, the DTI color map and fractional anisotropy (FA) atlases. The anatomical coordinates of individual axial slices are listed on the bottom of the figure.

To study brain morphological shape, we started with the construction of a neonatal T2-weighted atlas based on our own dataset using an atlas generation procedure [7]. In the first iteration of the atlas generation, the JHU neonate brain single-subject atlas (resolution: 0.6×0.6×0.6 mm^3^, http://lbam.med.jhmi.edu/) (Oishi et al., 2011) was used as initial atlas, where each subject’s T2-weighted image was aligned to via affine and nonlinear large deformation diffeomorphic metric mapping (LDDMM) [8] transformations. The image obtained by averaging the deformed images of individuals was considered as a new atlas. In the second iteration of the atlas generation, we then considered this new atlas as initial atlas and aligned all individual subjects to it via affine and LDDMM transformations. Three iterations were run to obtain the final T2-weighted neonatal atlas (**Figure 1A**) as the intensity change of the atlases obtained from the second and third iterations was less than 5%. The T2-weighted atlas generated using this procedure was used as a common atlas space for the following brain shape analysis. The T2-weighted MRI atlas is available for download at http://www.bioeng.nus.edu.sg/cfa/atlas/infant/index.html.

For studying brain shape, all subject T2-weighted images were first linearly aligned to our T2-weighted neonatal atlas using linear transformation (translations, rotations and scales) to ensure that the global orientation and size of individual brains are normalized. The brain shape of individuals was studies through its LDDMM transformation referenced to the atlas. The Jacobian determinant of the LDDMM transformation in the logarithmic scale was computed in the local coordinates of the atlas for statistical shape comparison across the ethnic groups. We shall refer to the logarithmic scale of the Jacobian determinant as the *deformation map* in the rest of the paper. Its value represents the ratio of each subject’s brain volume to the atlas volume in the logarithmic scale: i.e. positive values correspond to the expansion of a subject’s brain relative to the atlas at a particular location, while negative values denote the compression of subject’s brain relative to the atlas. The deformation map particularly measures the shape difference between the subject and the atlas in regions with high image contrast, such as the boundaries between the gray and white matters and between the subcortical structures and the white matter.

### DTI Analysis

DWIs were first corrected within individual subjects for motion and eddy current distortions using affine transformation to the image without diffusion weighting. Using multivariate least-square fitting, six elements of the diffusion tensor were then determined, from which fractional anisotropy (FA) was calculated. Similar to the above-mentioned morphological shape analysis, we constructed a DTI atlas and then aligned all the neonatal DTI to this atlas using LDDMM [8] for group analysis on ethnic differences in FA. In details, the FA image and the image without diffusion weighting of each subject were first aligned to those of the JHU neonate brain single-subject DTI atlas (resolution: 0.6×0.6×0.6 mm^3^, http://lbam.med.jhmi.edu/) [9] via affine and nonlinear LDDMM transformations. These affine and nonlinear transformations were then applied to DWIs. The mean DWIs were obtained by averaging the corresponding DWIs of individuals. The mean FA image was computed using the mean DWIs. These mean DWIs and FA were considered as a new atlas. This procedure was repeated three times. The mean DWIs and FA at the last iteration were used as the final atlas (**Figure 1B and 1C**) and used as a common anatomical space with which images were subsequently aligned for voxel-based analysis described in the next section. FA was obtained from the tensor calculation of DWIs aligned to the atlas. The DTI atlas is available for download at http://www.bioeng.nus.edu.sg/cfa/atlas/infant/index.html.

### Statistical Analysis

Analysis of Covariance (ANCOVA) with population (Chinese, Malay, Indian) as the main factor was used to examine population differences in continuous demographic variables and brain global size and morphology. For the brain measures, both post-gestational age and body weight on the MRI day were included as covariates in ANCOVA. Post-hoc analyses were carried out for the brain measures with significant group differences while Tukey’s tests were used for correcting multiple comparisons.

Voxel-based analysis was first performed to investigate population differences in the brain morphological shape and microstructure (FA) using SPM8. The deformation maps and FA images were smoothed with a Gaussian kernel with full width half maximum of 4 mm. ANCOVA with population (Chinese, Malay, Indian) as the main factor was performed at each voxel level when controlling for post-gestational age. The body weight on the MRI day was only included as covariate in morphological shape analysis. The ANCOVA tests for FA were performed in the white matter mask where the mean FA value is greater than 0.09. Regions of voxels with p≤0.001 and their cluster size greater than 50 voxels were considered as regions-of-interest (ROIs) with significant group differences.

Post-hoc analysis for pairwise group comparisons was performed for individual regions of interest (ROIs). These ROIs were the anatomical clusters identified from the above voxel-based analysis. The deformation map (or FA) values averaged across each ROI and used in the post-hoc analysis as dependent variables. Bonferroni correction was employed for correcting multiple comparison in the post-hoc analysis. The significance level for individual post-hoc tests was determined as 0.05 divided by the number of post-hoc tests examined.

Given evidence that prenatal exposure to alcohol and tobacco affect the developing brain’s structure and cognition [10], [11], we repeated the post-hoc analysis with prenatal tobacco exposure as covariate while excluding the subjects (n = 3) whose mother reported alcohol consumption during pregnancy. Since household income and ethnicity are related (p = 0.007, Table 1) in our sample, the post-hoc analysis was repeated with household income as a covariate.

Because fewer Indians participated in this study than did Chinese and Malays, we employed a randomization procedure to ensure that our statistical results were not influenced by unequal sample sizes across the ethnic groups. To do so, we first randomly chose the subsets of Chinese and Malays to match the sample size of the Indians and repeated our post-hoc analysis to investigate pairwise ethnic group difference involving the Indian group. We repeated this for one thousand times and computed the percentage of trials with significant ethnic group differences to demonstrate the repeatability of our findings using the equal sample sizes of Chinese, Malay, and Indian subjects.

## Results

### Demographic Information

Gender, birth weight, body weight on the MRI day and prenatal alcohol exposure did not differ among the Chinese, Malay, and Indian groups in this study (**Table 1**). However, differences among the three groups were found in gestational age (p = 0.048), post-gestational age on the MRI day (p = 0.050), monthly household income (p = 0.007), and prenatal tobacco exposure (p<0.001) (**Table 1**).

### Brain Global Size and Morphology

Among the Chinese, Malay, and Indian neonates, no group differences were found in the total brain volume, AC-PC length, the three-dimensional lengths of the brain, brain length to width ratio, and the brain height to width ratio (**Table 2**). However, the brain length to height ratio differed between the three groups (p = 0.022). Post-hoc analysis further revealed that the brain length to height ratio of the Malay neonates was significantly smaller than that of the Indian neonates (p = 0.002), suggesting that the brain of the Malay neonates is rounder than that of Indian neonates. This group difference remained significant after controlling for household income (p = 0.004) and prenatal tobacco exposure (p = 0.04).

**Table 2:** Brain global size and morphology.

	Chinese Mean (SD)	Malay Mean (SD)	Indian Mean (SD)	F_2,174_	p-value
**Global Size**					
**Total Brain Volume, ×10^5^** **mm^3^**	5.89 (0.51)	5.80 (0.46)	5.83 (0.56)	0.19	0.831
**AC-PC Length, mm**	20.36 (0.81)	20.16 (0.85)	20.07 (0.84)	1.12	0.327
**Length, mm**	115.35 (4.18)	114.32 (4.11)	115.45 (0.84)	1.65	0.195
**Width, mm**	95.49 (3.91)	95.33 (3.98)	94.45 (4.13)	0.40	0.672
**Height, mm**	89.07 (3.44)	89.26 (3.75)	87.79 (3.22)	1.89	0.154
**Global Morphology**					
**Length/Width**	1.21 (0.05)	1.20 (0.04)	1.22 (0.06)	2.29	0.105
**Length/Height**	1.30 (0.06)	1.28 (0.06)	1.32 (0.06)	3.91	**0.022** *
**Height/Width**	0.93 (0.05)	0.94 (0.05)	0.93 (0.05)	0.51	0.602

* Post-hoc analysis revealed that the length to height ratio of the Malay neonatal brain was significantly smaller than that of Indian (p = 0.002).

Abbreviation: SD – standard deviation.

### Brain Morphological Shape

**Figure 2:**
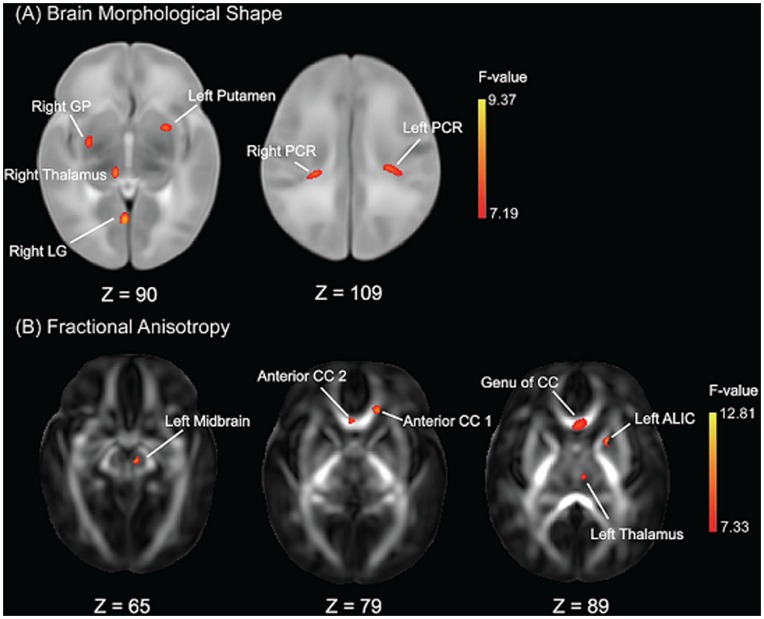
Statistical Maps of ethnic group difference in brain morphological shape (A) and fractional anisotropy (B). The anatomical coordinates of each axial slice are given at the bottom of each panel. Abbreviations: LG – lingual gyrus; PCR – posterior corona radiata; GP – globus pallidus; ALIC – anterior limb of internal capsule; CC – corpus callosum.

After controlling for post-gestational age and the body weight on the MRI day, voxel-based analysis revealed morphological shape differences among the Chinese, Malay, and Indian neonates in the left putamen, right thalamus, right globus pallidus (GP), right lingual gyrus (LG), left and right posterior corona radiata (PCR) regions (**Figure 2A** **,** **Table 3**).

**Figure 3:**
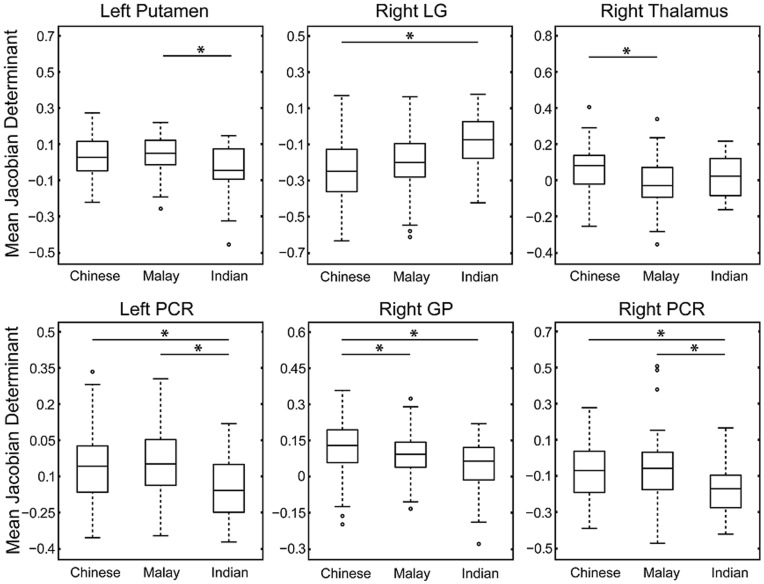
Boxplots of mean deformation within each anatomical region extracted from voxel-based analysis on the brain morphological shape (see Figure 2A). The significant pairwise group differences are indicated using black asterisk.

**Figure 3** illustrates boxplots of mean values for each of the above anatomical regions in the groups of Chinese, Malay, and Indian. The post-hoc analysis on the mean deformation within each ROI revealed that when compared to Indian neonates, Chinese neonates showed significant volume change in the abovementioned anatomical regions except the left putamen and right thalamus after adjusting post-gestational age and the body weight on the MRI day (**Table 4**). Additionally, Chinese neonates also showed significant volume expansion in the right thalamus and right GP when compared to Malay neonates. Lastly, when compared to Indian neonates, Malay neonates showed significant volume expansion in the left putamen, left and right PCR after adjusting for post-gestational age and weight on the MRI day (**Table 4**). Most of these findings remained significant regardless of adding either household income or prenatal tobacco exposure as covariates (**Table 4**). The only exception is the comparison of the left putamen between Malay and Indian neonates where the result became insignificant after adjusting for prenatal tobacco exposure as covariates.

**Table 3:** Population group comparisons in brain morphological shape and fractional anisotropy.

Brain Regions	ClusterSize	Peak F-value	Peakp-value	x	y	z
**Brain Morphological Shape**						
**Left Putamen**	420	8.51	0.0003	118	130	74
**Right Lingual Gyrus (LG)**	465	9.37	0.0001	86	58	74
**Right Thalamus**	342	9.03	0.0002	78	94	76
**Left Posterior Corona Radiata (PCR)**	422	8.78	0.0002	118	96	106
**Right Globus Pallidus (GP)**	217	8.71	0.0002	58	118	74
**Right Posterior Corona Radiata (PCR)**	256	8.32	0.0004	62	94	108
**Fractional Anisotropy**						
**Left Anterior Limb of Internal Capsule**	261	12.81	<0.0001	113	130	85
**Anterior Corpus Callosum (region 1, CC 1)**	176	10.24	<0.0001	107	157	78
**Anterior Corpus Callosum (region 2, CC 2)**	87	10.11	<0.0001	87	148	78
**Left Midbrain**	267	9.83	0.0001	95	110	63
**Genu of Corpus Callosum (CC)**	314	9.69	0.0001	90	145	90
**Left Thalamus**	72	8.76	0.0003	95	103	88

Furthermore, by randomly reducing the sample size of the Chinese and Malay neonates to match that of the Indian neonate, these findings listed in Table 3 can be found 88.7% among 1000 repeated analyses for the comparisons between the Chinese and Indian groups and 79.6% between the Malay and Indian groups.

### White Matter Microstructure

Voxel-based analysis revealed six anatomical clusters with significant population difference in FA after controlling for post-gestational age on the MRI day. As illustrated in **Figure 2B**, these clusters are located in the left anterior limb of internal capsule (LIC), anterior corpus callosum (CC), left midbrain, genu of corpus callosum and left thalamus regions (**Table 3**).

**Figure 4:**
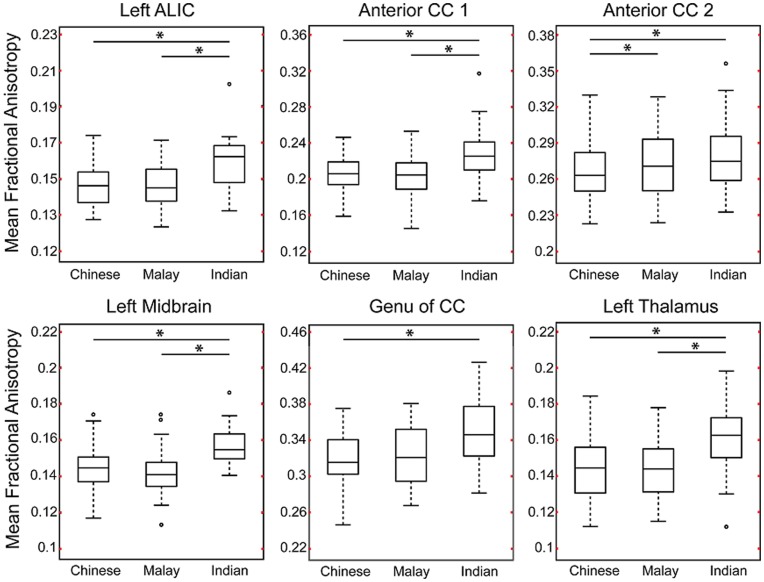
Boxplots of mean fractional anisotropy (FA) within the anatomical regions extracted from voxel-based analysis on FA (see Figure 2B). The significant pairwise group difference is indicated using black asterisk.

**Figure 4** illustrates boxplots of mean FA values across the six anatomical clusters in the groups of Chinese, Malay, and Indian. The ROI-based analysis on the mean FA value revealed that Chinese neonates had significantly lower mean FA only in anterior CC 2 when compared to Malay neonates after controlling for post-gestational age at MRI (**Table 4**). This result remained unchanged even after controlling for household income or prenatal tobacco exposure (**Table 4**). Additionally, Chinese neonates had smaller FA values in all six clusters when compared to Indian neonates after controlling for post-gestational age on the MRI day (**Table 4**). These group differences in the mean FA between the Chinese and Indian neonates remained unchanged even after controlling for household income or prenatal tobacco exposure (**Table 4**), except in the anterior CC2 region after additionally controlling for household income. Lastly, when compared to Indian neonates, Malay neonates had significant FA reductions in four clusters, including the left ALIC, anterior CC 1, left midbrain, and left thalamus, after controlling for post-gestational age at MRI (**Table 4**). Most of these findings remained unchanged even after controlling for household income or prenatal tobacco exposure (**Table 4**). However, the group difference in the mean FA of left ALIC and anterior CC 1 between Malay and Indian was no longer significant when prenatal tobacco exposure was added as covariate.

**Table 4:** P-values for pairwise group comparisons on brain morphological shape and fractional anisotropy within each brain region listed in Table 3.

Brain Regions	Chinese vs Malay			Chinese vs Indian			Malay vs Indian		
	Model1	Model2	Model3	Model1	Model2	Model3	Model1	Model2	Model3
**Brain Morphological Shape**									
**Left Putamen**	0.064	–	–	0.015	–	–	<0.001*	<0.001*	0.010
**Right LG**	0.012	–	–	<0.001*	<0.001*	<0.001*	0.012	–	–
**Right Thalamus**	<0.001*	<0.001*	<0.001*	0.115	–	–	0.162	–	–
**Left PCR**	0.812	–	–	<0.001*	0.005*	<0.001*	<0.001*	<0.001*	<0.001*
**Right GP**	0.005*	0.008*	0.002*	<0.001*	<0.001*	<0.001*	0.039	–	–
**Right PCR**	0.154	–	–	<0.001*	0.002*	<0.001*	0.002*	<0.001*	0.003*
**Fractional Anisotropy**									
**Left ALIC**	0.066	–	–	<0.001*	<0.001*	0.007*	0.003*	0.006*	0.042
**Anterior CC 1**	0.490	–	–	0.001*	0.003*	0.001*	0.004*	0.007*	0.016
**Anterior CC 2**	0.001*	0.003*	0.005*	0.003*	0.022	0.008*	0.535	–	–
**Left Midbrain**	0.426	–	–	<0.001*	<0.001*	0.003*	<0.001*	<0.001*	0.003*
**Genu of CC**	0.017	–	–	<0.001*	<0.001*	<0.001*	0.033	–	–
**Left Thalamus**	0.678	–	–	<0.001*	<0.001*	<0.001*	<0.001*	<0.001*	0.003*

Abbreviations: LG – lingual gyrus; PCR – posterior corona radiata; GP – globus pallidus; ALIC – anterior limb of internal capsule; CC – corpus callosum.

Model 1: deformation ∼ ethnicity + post-gestational age + body weight on the MRI day; FA ∼ ethnicity + post-gestational age;

Model 2: deformation ∼ ethnicity + post-gestational age + body weight on the MRI day + household income; FA ∼ ethnicity + post-gestational age + household income;

Model 3: deformation ∼ ethnicity + post-gestational age + body weight on the MRI day + prenatal tobacco exposure FA ∼ ethnicity + post-gestational age + prenatal tobacco exposure.

* group difference at the significance level of 0.0083 (0.05/6; Bonferroni correction for multiple ROIs).

- analysis was not performed since no group difference was found using model 1. The significance level was chosen as 0.05/6 to correct for multiple comparisons.

Furthermore, by reducing the sample size of the Chinese and Malay neonates to match that of the Indian neonate, these findings listed in Table 4 can be found 90.3% among 1000 repeated analyses for the comparisons between the Chinese and Indian groups and 60.3% between the Malay and Indian groups.

## Discussion

To our knowledge this is the first large scale study examining differences in global brain size, morphological shape, and white matter microstructure among Chinese, Malay and Indian neonates using both structural MRI and DTI. Although the global brain size was similar among the three populations of neonates in terms of volume, we found that the brain of Indian neonates tended to be more elongated than that of Malay neonates in the anterior-posterior axis relative to the superior-inferior axis. Moreover, analyses of brain morphological shape and white matter microstructure revealed that region-specific anatomical variations of the brain among Chinese, Malay, and Indian neonates were relatively sparse but mostly occurred in the spinal-cerebellar and cortical-striatal-thalamic neural circuits associated with sensorimotor functions. These anatomical variations were mainly due to group differences of Indian neonate with Chinese and/or Malay neonates.

Previous studies of head circumference have suggested difference in brain size between Asian American, white, and black children from birth through childhood [12]. Lee et al. [2] and Tang et al. [3] employed structural MRI and demonstrated substantial variations in the three dimensional lengths between Chinese/Korean and Caucasian adults. Unlike the aforementioned findings, we found no differences in global brain size among the three Asian populations at birth. However, the three Asian populations showed the distinct pattern of brain global shape at birth particularly in the anterior-posterior axis relative to the superior-inferior axis, which may partly contribute to the region-specific structural differences reported in our study. This may or may not persist through later life, which needs the follow-up investigation.

The population-related brain regions highlighted in our study include the spinal-cerebellar and cortical-striatal-thalamic neural circuits that are key anatomical substrates associated with sensorimotor functions. During the second trimester, the commissural fibers [13], projection fibers (ALIC and PCR) and inferior cerebellar penducle (ICP, in the midbrain) are visible using tractography in *ex-vivo* DTI [14], [15]. Despite some of these tracts, such as the CC, not reaching full maturation until adulthood, the majority are in the limited myelinated brain regions at birth, including brainstem, cerebellar white matter and internal capsule with extensions to the thalamus and basal ganglia [16]. These tracts are involved in several neural circuits supporting basic brain functions at birth. For example, the ICP is posterior to the superior cerebellar peduncle from which the thalamic radiations emanate and convey information from the spinal cord to the cerebellum. As such, the ICP helps to integrate sensory inputs with motor functions to allow for balance and posture maintenance. Second, the ALIC is a major cortico-subcortical white matter bundle. It contains fibers running from the thalamus to the basal ganglia as well as connecting the thalamus to the frontal lobe, which is responsible for regulation of sensorimotor functions. Third, the PCR is the continuation of the posterior limb of the internal capsule as it makes its way to sensorimotor cortex in and near the central sulcus. In addition, the PCR as part of the cortical-striatal-thalamic circuit forms the biological basis of fetal consciousness [17]. Finally, the formation of the CC is relatively more advanced in the frontal lobe than other brain regions during the fetal stage, which may further emphasize the importance of inter-hemispherical communication in the frontal region. Taken together, these findings underscore the importance of a developmental approach to the examination of population differences, and utilizing sensitive methodologies such as structural MRI and DTI. However, because DTI is still a relatively new technique, the functional significance of these findings is unclear. Relatively greater FA in regions such as ALIC and CC at birth may indicate greater coherence in fibers organization and greater proliferation of oligodendrocytes prior to myelin ensheathment [18].

Thus far, there is no evidence of varying sensorimotor functions among different ethnic groups, particularly among Asian populations. Also, it remains unclear whether the anatomical differences found at birth in our study have later consequences. Nevertheless, there are reports of population differences in the frequency and symptomatology of neurodevelopmental disorders that involve structural differences in regions found in our study, such as schizophrenia, cerebral palsy and attention deficit hyperactivity disorder (ADHD). Lim et al. [19] report differences in severity of symptomatology among Chinese, Malay, and Indian adults with first-episode schizophrenia spectrum disorder. With converging evidence that schizophrenia is a neurodevelopmental disorder with disruption of the thalamic-cortical circuit [20], we speculate that the brain variations at birth may relate to the population differences in the presentation of schizophrenia. Similarly, differences in prevalence and severity of symptoms have been reported between populations for cerebral palsy and ADHD. The thalamus, basal ganglia, and ALIC are anatomical substrates vulnerable to brain damage (e.g., cerebral palsy) in the perinatal and early postnatal period [21], [22], [23] and also show anatomical abnormalities in ADHD [24]. The thalamus [25], putamen [26], [27] and lingual gyrus [28] have been implicated in both schizophrenia and ADHD in Caucasian populations. Future research on the direct link between brain anatomical variations at birth and the pattern of presentation of behavioral and neuropsychiatric disorders across different populations could help to identify potential etiologies and inform more appropriate intervention practices for a specific ethnic population early in life.

A large body of research focuses on seeking etiological risk factors, such as socioeconomic status, smoking, alcohol, education, health service and so on, that contribute to population differences in the prevalence of neurodevelopmental disorders [29], [30]. Our findings of population differences in brain structures remained largely unchanged when major prenatal factors, including household income, smoking and alcohol exposures, were taken into account.

This study has a number of limitations. First, there was the lack of the examination on sensorimotor functions at birth in our cohort. Hence, we could not report the variation of sensorimotor functions and its associations with the ethnic-related brain structural differences among the three ethnic groups in this study. However, our study presented the first evidence of brain anatomical differences among Chinese, Malay, and Indian neonates. Second, in our study the sample size of the Indian neonates is smaller than those of the Chinese and Malays partially due to the fact that the Indian is one of minority populations in Singapore. We performed randomization analysis in which the sample sizes of the three ethnic groups were matched. Our findings were highly repeatable, suggesting negligible effects on our findings due to the small sample size in the Indian group.

In conclusion, our study has demonstrated population-related differences in brain morphological shape and white matter microstructure but not global size among the three Asian populations at birth. The population-related brain regions highlighted in our study include the spinal-cerebellar and cortical-striatal-thalamic neural circuits that are anatomical substrates mostly associated with sensorimotor functions.
